# From Global Insights to Local Action: Bridging Vaccine Design and Manufacturing Gaps in H5N1 Pandemic Readiness

**DOI:** 10.3390/vaccines14060519

**Published:** 2026-06-10

**Authors:** María Alicia Delfino, Jimena Borgo, Luciano Chaneton, Natacha Cerny, Augusto Ernesto Bivona, Pierre Gsell, Fernando Lobos, Ike James, Martin Friede, German Sánchez Alberti, Andrés Sánchez Alberti

**Affiliations:** 1 Facultad de Medicina, IMPAM (UBA-CONICET), Universidad de Buenos Aires, Buenos Aires 1121, Argentina; 2 Sinergium Biotech, Buenos Aires 1619, Argentina; 3 World Health Organization, 1211 Geneva, Switzerland; 4 Medicines Patent Pool, 1202 Geneva, Switzerland

**Keywords:** pandemic preparedness, pandemic influenza vaccine, mRNA vaccine, neuraminidase antigen, bivalent influenza vaccine, technology transfer

## Abstract

The global expansion of highly pathogenic avian influenza A (H5N1), particularly the clade 2.3.4.4b lineage, has renewed urgent concerns about its pandemic potential in the context of its ongoing panzootic expansion and increasing cross-species transmission. Despite decades of preparedness initiatives, critical technological and structural gaps persist, especially in low- and middle-income countries (LMICs), where both vaccine access and sustainable manufacturing capacity remain limited. In this perspective, we examine key lessons from past influenza pandemics and global preparedness strategies, including the Global Action Plan for Influenza Vaccines, highlighting persistent challenges related to sustainable manufacturing capacity and equitable vaccine access. Additionally, we examine the potential of messenger RNA (mRNA) vaccine platforms to address these limitations, given their rapid design, scalable manufacturing, and adaptability to emerging pathogens. Moreover, we examine the role of neuraminidase (NA) as a complementary antigen capable of broadening immune protection and reducing viral transmission. Finally, we describe recent advances in Latin America, focusing on Argentina’s participation in the mRNA Technology Transfer Programme co-led by the World Health Organization (WHO) and the Medicines Patent Pool (MPP), as a model for strengthening regional manufacturing capacity and contributing to global pandemic preparedness. Together, these elements indicate that effective H5N1 pandemic preparedness will require the integration of improved antigen design, flexible mRNA platforms, and sustainable regional manufacturing systems aligned with global procurement strategies.

## 1. Introduction

Influenza is an infectious respiratory disease that continues to pose a substantial global health burden, causing high levels of morbidity and mortality each year [1]. Although characterized by annual seasonal epidemics, influenza viruses also give rise to sporadic and unpredictable pandemics involving novel influenza A strains of zoonotic origin [2].

Influenza A viruses are found in wild birds, especially waterfowl and seabirds, which serve as natural reservoirs. Spillover events, particularly due to contact between wild and domestic birds, can lead to severe poultry outbreaks [3].

These animal reservoirs harbor a wide diversity of viral subtypes, defined by their hemagglutinin (Hx) and neuraminidase (Ny) surface proteins. The influenza virus has a negative sense RNA genome composed of eight segments, which can be exchanged between viral strains after co-infection of the same cell, ultimately producing a novel variant. This phenomenon increases viral diversity, against which human populations may have little or no pre-existing immunity, leading to the emergence of viruses derived from animal strains that may cause human pandemics [2,4].

Since the early 20th century, influenza A has caused four major pandemics. In 1918, the H1N1 subtype was responsible for the worst pandemic in recorded history, causing between 20 and 50 million deaths. Subsequent pandemics, including H2N2 in 1957 and H3N2 in 1968, caused an estimated 1–4 million deaths each, while the 2009 H1N1 pandemic resulted in approximately 100,000–400,000 deaths globally [5,6]. Notably, at least the last three pandemics were caused by reassorting viruses originating from both animal and human seasonal influenza A [7]. These events underscore the persistent risk posed by zoonotic influenza viruses and the importance of preparedness strategies capable of responding to emerging threats.

Highly pathogenic avian influenza A (HPAI) viruses have been responsible for widespread outbreaks in birds, causing major economic losses and posing a continued threat to animal and human health [2]. Among these, the H5N1 subtype has emerged as a particularly concerning pathogen. First identified in humans in Hong Kong in 1997, H5N1 has since re-emerged and spread across Asia, Africa, Europe, and, more recently, the Americas, affecting a wide range of avian and mammalian species [5,8]. As of early 2026, nearly 1000 human cases have been reported across 25 countries, with a case fatality rate of approximately 50% [9].

Since 2021, the clade 2.3.4.4b lineage has driven an unprecedented global panzootic, infecting diverse bird populations and an increasing number of mammalian hosts [10]. While most human infections have historically been associated with direct exposure to infected animals, recent evidence suggests that this lineage may possess an increased capacity for mammal-to-mammal transmission [11,12]. The expanding host range and geographic spread of H5N1 significantly increase opportunities for viral adaptation to humans, reinforcing concerns about its pandemic potential. In this context, the ongoing H5N1 panzootic provides a critical case study for assessing current preparedness strategies and identifying persistent gaps in the global response.

To address the risks associated with pandemic influenza, substantial international efforts have been undertaken over the past two decades. The rapid spread and diversification of H5N1 since 2003 forced the implementation of global actions that helped shape public health response planning. This marked a turning point in pandemic preparedness by helping to identify key structural and technological gaps. The “World Health Organization (WHO) global influenza preparedness plan”, published in 2005 to assist member states in the response planning, outlined the recommended actions into five main categories: (1) planning and coordination; (2) situation monitoring and assessment; (3) prevention and containment; (4) health system response; and (5) communication [11]. This was later updated to incorporate lessons from the 2009 H1N1 influenza pandemic [12]. Following the COVID-19 global emergency, an integrated approach was designed to strengthen pandemic preparedness against respiratory pathogens in alignment with the International Health Regulations 2005 (IHR) and the WHO Guidance on Preparing for National Response to Health Emergencies and Disaster [13].

Despite these advances, important gaps remain at both the technological and structural levels. Current influenza vaccine strategies continue to rely predominantly on hemagglutinin-focused approaches, which may limit the breadth and durability of immune protection. At the same time, global manufacturing capacity remains unevenly distributed and difficult to sustain outside pandemic periods, particularly in low- and middle-income countries (LMICs). These challenges highlight that effective preparedness depends not only on surveillance and planning frameworks, but also on the availability of adaptable vaccine technologies and resilient, regionally embedded production systems.

In this perspective, we examine key gaps in influenza pandemic preparedness and explore how emerging approaches—including mRNA vaccine platforms, complementary antigen strategies such as neuraminidase, and regionally embedded manufacturing models—can address these limitations. We place particular emphasis on recent advances in Latin America as a case study illustrating how global strategies can be aligned with local implementation. We argue that effective preparedness for emerging threats such as H5N1 will require the integration of improved antigen design, flexible and scalable vaccine platforms, and sustainable regional manufacturing systems, supported by coordinated global procurement mechanisms.

## 2. Technological and Strategic Gaps in Influenza Pandemic Preparedness

### 2.1. The Global Action Plan for Influenza Vaccines (GAP): Achievements and Persistent Gaps

The COVID-19 pandemic exposed long-standing global inequities in access to medical countermeasures during health emergencies, particularly affecting LMICs [14]. Similar challenges were already evident during the 2005 H5N1 outbreak and repeated during the 2009 H1N1 pandemic, when many LMICs called for technology transfer to enable local vaccine production. In response, the World Health Organization launched the Global Action Plan (GAP) for Pandemic Influenza Vaccines, which aimed to reduce global supply gaps by transferring manufacturing know-how, strengthening regulatory capacity, and supporting end-to-end production in LMICs [15,16]. Between 2006 and 2016, the GAP programme supported manufacturers in 14 countries, nine of which achieved licensure of locally produced influenza vaccines, increasing global pandemic influenza vaccine capacity to approximately 900 million doses annually—nearly one quarter of the estimated global capacity [16,17].

However, the GAP experience demonstrated that the establishment of manufacturing capacity alone does not guarantee sustained pandemic preparedness. Several recipient manufacturers subsequently scaled down, mothballed, or closed their facilities due to insufficient demand for seasonal influenza vaccines to sustain operations during inter-pandemic periods [16,18]. This challenge was exacerbated by the technological characteristics of egg-based influenza vaccine production, which offers limited flexibility for product diversification and requires long lead times for scale-up, particularly due to dependence on specialized egg supply chains [18,19]. As a result, facilities designed to respond rapidly to pandemic-scale demand were often economically unviable outside pandemic periods, leading to erosion of infrastructure, workforce expertise, and supply chain readiness [18,20]. These structural limitations underscore the importance of sustainable market demand, platform flexibility, and manufacturing integration beyond single-pathogen preparedness strategies.

The GAP experience thus provides critical lessons for current efforts to decentralize vaccine manufacturing, including mRNA technology transfer initiatives (Table 1). In the absence of sustainable, non-pandemic, routine product pipelines and reliable procurement mechanisms, newly established capacity may face a similar decline, undermining long-term regional and global preparedness [21,22]. These limitations stem partly from the inherent constraints of conventional vaccine manufacturing technologies. Conventional influenza vaccines are primarily produced using embryonated chicken eggs, a manufacturing approach that has been employed for more than seven decades and remains the most widely used platform worldwide. Egg-based production is supported by well-established infrastructure, extensive regulatory experience, and relatively low production costs, making it suitable for large-scale seasonal vaccine manufacturing. In this process, candidate vaccine viruses are inoculated into fertilized eggs and propagated over several days before viral harvest and purification. Egg-based systems have historically demonstrated robust productivity and have contributed substantially to global influenza prevention programs. In addition, the production process incorporates multiple purification and inactivation steps that traditionally reduce the risk of microbial contamination. Nevertheless, this platform presents important limitations. Manufacturing timelines are relatively long and highly dependent on the continuous availability of large numbers of pathogen-free fertilized eggs (1–3 eggs per vaccine dose), which may become problematic during avian disease outbreaks or pandemic emergencies. Egg-based production also lacks flexibility, as vaccine strain selection must occur months before the influenza season to accommodate manufacturing requirements. Furthermore, propagation of influenza viruses in eggs can promote the emergence of egg-adaptive mutations, leading to antigenic alterations that may reduce the similarity between vaccine antigens and circulating viruses and potentially compromise vaccine effectiveness. Egg-derived vaccines may also contain residual egg proteins, which need additional purification steps and may represent a concern for individuals with severe egg allergies, although current vaccines are considered safe for most allergic patients [23,24,25].

In contrast, cell-culture-based technologies utilize mammalian cell lines, such as MDCK or Vero cells, for viral propagation and have emerged as a promising alternative for influenza vaccine production. Viruses propagated in cell culture generally maintain antigenic characteristics that more closely resemble circulating strains, potentially improving vaccine strain matching. Additionally, cell-based systems provide enhanced batch consistency, improved traceability, and reduced dependence on egg supplies and egg-derived components. However, the implementation of cell-based platforms also involves substantial technical and regulatory challenges. Optimizing virus harvest yields and downstream purification strategies capable of generating high quantities of properly folded and immunogenic influenza antigens remain a complex task. Moreover, vaccine seed viruses frequently require additional adaptation for efficient replication in mammalian host cells, which can be difficult for certain strains. Regulatory requirements are also stringent, as qualified cell banks must be extensively tested to confirm the absence of contaminant agents and comply with tumorigenicity and oncogenicity standards while maintaining strict sterility and low bioburden conditions. These quality-control procedures are often labor-intensive, technically demanding, and time-consuming. Despite these challenges, the scalability, flexibility, and improved antigenic fidelity associated with cell-based manufacturing platforms position them as increasingly important alternatives for both seasonal and pandemic influenza vaccine development [25,26,27,28].

### 2.2. The Need for Faster and More Flexible Platforms: The Case for mRNA

Addressing the limitations of traditional vaccine platforms requires the adoption of technologies capable of rapid adaptation, scalable production, and broader applicability across multiple pathogens. The messenger RNA (mRNA) platforms have emerged as a promising solution to these challenges since the same infrastructure and process can potentially be used for multiple different routine and outbreak vaccines as well as therapeutic applications, increasing the feasibility of maintaining sustainable manufacturing.

mRNA vaccines, which encode antigenic proteins capable of eliciting immune responses, have been studied since the 1990s [29,30]. Interest in this technology has grown significantly due to its ability to encode diverse antigens using scalable, cell-free production processes [31]. Unlike conventional vaccine platforms, mRNA production does not require cell culture or egg-based systems, enabling rapid design and manufacturing once a pathogen’s genetic sequence is known.

Early development of mRNA vaccines faced several technical challenges, including the intrinsic instability of RNA, inefficient delivery to target cells, and unintended activation of innate immune responses. Over time, these limitations have been largely addressed through advances in RNA chemistry and delivery systems whilst high production costs, storage and distribution conditions (−80 °C) are currently the main disadvantages associated with this vaccine platform. Ongoing development efforts are working to address these constraints through improved formulations, stabilization technologies, and cold-chain solutions [32,33,34,35].

mRNA vaccine constructs, like endogenous mRNA, typically contain several sections that include, from 5′ to 3′ a 5′ cap, a 5′ untranslated region (5′ UTR), an open reading frame (ORF) that encodes the antigen, a 3′ UTR and a poly(A) tail. Optimization strategies have focused on improving translational efficiency, reducing undesired innate immune activation, and increasing mRNA stability. Unmodified single-stranded RNA is recognized by pattern recognition receptors such as TLR3, TLR7, TLR8, and RIG-I, leading to type I interferon responses. To mitigate this, modified nucleosides, such as pseudouridine and N1-methylpseudouridine are incorporated to reduce immune recognition [36]. Additionally, codon optimization strategies, including the use of GC-rich codons to reduce uridine content, help limit activation of TLR7 and TLR8 [37]. Stability and delivery have also been significantly improved through the development of advanced delivery systems, particularly lipid nanoparticles (LNPs), which enable efficient intracellular delivery and protection of the mRNA molecule [38,39].

The COVID-19 pandemic demonstrated the full potential of mRNA for rapid vaccine development. Notably, the first human administration of an mRNA vaccine occurred just 64 days after publication of the SARS-CoV-2 genome sequence [32]. As of 2026, six mRNA and three self-amplifying RNA (saRNA) vaccines have been authorized for COVID-19, while an mRNA vaccine against the respiratory syncytial virus (RSV) infection has also been approved for human use [40]. In contrast, in the last influenza pandemic, vaccines were not available on time to contain the first waves of infection [41]. This is partially related to the fact that the manufacturing process for influenza virus vaccines has remained largely unchanged for many decades. While most influenza vaccines are manufactured using an egg-based platform, cell culture- and recombinant protein-based vaccine platforms are also available.

A key challenge specific to the conventional egg- and cell culture-based vaccine platforms is the approximate 6-month production time after initial vaccine composition recommendations, allowing time for an antigenically divergent clade to predominate and potentially cause a mismatch to vaccine strain compositions [42]. Recent regulatory progress, including the European Medicines Agency (EMA) recommendation for marketing authorization of the first combined mRNA vaccine against COVID-19 and seasonal influenza, represents an important step forward in the implementation of this technology against seasonal and pandemic influenza virus [43].

Beyond speed, the strategic value of mRNA lies in its platform nature. The same manufacturing infrastructure can be applied across multiple vaccine targets and therapeutic indications, enabling diversification of product pipelines. This directly addresses one of the key limitations identified in the GAP experience—namely, the lack of sustainable demand and flexibility in manufacturing systems during inter-pandemic periods. By supporting both pandemic and non-pandemic applications, mRNA platforms can help maintain operational readiness, workforce expertise, and supply chain continuity over time.

Taken together, mRNA technology addresses several of the critical gaps in influenza pandemic preparedness, including slow production timelines, limited platform flexibility, and lack of sustainable manufacturing models. Its capacity for rapid design, scalable production, and cross-pathogen applicability positions mRNA as a central component of next-generation pandemic preparedness strategies as summarized in Table 1. However, many challenges remain ahead as discussed below.

### 2.3. The Importance of Neuraminidase (NA) as a Complementary Antigen

Influenza vaccine development has historically prioritized immunity directed against hemagglutinin (HA). Since the introduction of seasonal influenza vaccines in 1940, licensed formulations have been primarily standardized based on their HA content. Consequently, the quantity and quality of the neuraminidase (NA) present in these formulations are not strictly controlled [39]. This regulatory focus has led to a significant gap in NA-based immunity among vaccine recipients [40]. The historical emphasis on HA is largely due to its essential role in viral entry and its predominance—comprising approximately 80% of total surface glycoproteins. Furthermore, neutralizing antibodies directed against HA remain the primary mediators of protection against infection and clinical illness [41].

Beyond HA, NA represents a vital target with a distinct mechanism of action as a sialidase enzyme [42]. By enzymatically removing sialic acids from cell surface receptors and nascent virions, NA facilitates the release of new progeny. This mechanism prevents the virus from being trapped on the host cell, a critical step that NA-directed antibodies can effectively interrupt [43,44]. While the abundance of NA on the viral surface is lower compared to HA, with an HA:NA ratio of 8:1, natural infection induces both homologous and cross-reactive anti-NA responses that can contribute to protection [45,46,47].

**Figure 1:**
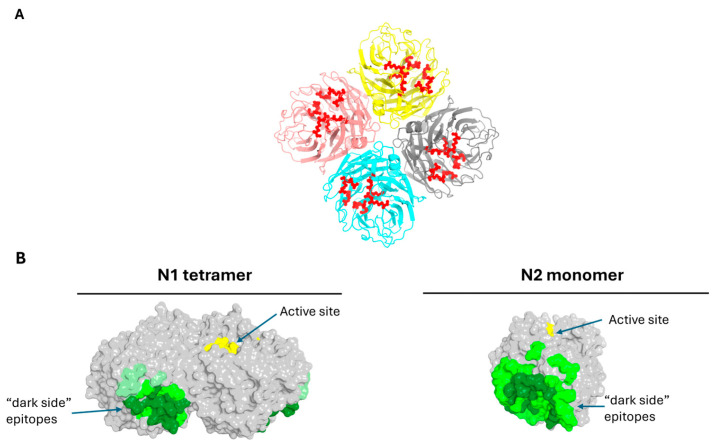
Neuraminidase tetramer architecture and conservation of “dark side” epitopes. (**A**) Top view of quaternary structure of N1 neuraminidase tetramer (PDB 8E6J) shown in cartoon representation. Each monomer adopts a six-bladed propeller fold and is colored distinctly (cyan, gray, yellow, salmon). Red spheres indicate the lateral catalytic sites positioned on the sideward face of each head domain, optimally oriented for sialic acid cleavage from nearby glycoproteins. (**B**) Highly conserved and cross protective regions of N1 and N2 subtypes. Left: side view of N1 neuraminidase tetramer (PDB 8E6J) showing epitopes of mAbs 2H08 and 3H03 [48]. N2 neuraminidase monomer (PDB 8EZ3) showing epitopes of mAbs 3C08, 3A10, and 1F04 [52]. Both subtypes harbor analogous epitope regions on the face opposite their catalytic sites—the “dark side” of neuraminidase. Color scheme: Dark green, core epitope residues contacted by multiple monoclonal antibodies; light green, antibody-specific contact residues; yellow, catalytic site; gray, remaining neuraminidase surface.

Structurally, the NA catalytic head is a box-shaped tetramer composed of four monomers, each adopting a six-bladed propeller fold stabilized by disulfide bonds [48]. Each monomer contains a lateral, sideward-facing catalytic site—an orientation optimized to cleave sialic acids from nearby glycoproteins [49] (Figure 1A). Evidence demonstrates that the tetrameric form of NA is required to induce protective antibody responses; monomeric or misfolded forms often fail to elicit neuraminidase-inhibiting (NAI) antibodies capable of providing protection against lethal viral challenge [50,51].

Furthermore, structural studies have identified conserved regions that are less prone to immune-driven selective pressure, making them potential targets for eliciting heterosubtypic immunity. These include the conserved active site, targeted by recently identified broadly neutralizing antibodies such as CAV-F6 and CAV-F34, which demonstrate protection against both seasonal and highly pathogenic avian H5N1 strains [53], and the ‘dark side’ epitopes located on the more shielded lateral and basal surfaces of the NA head [54,55] (Figure 1B). Recent cryo-electron microscopy studies by Hansen et al. [56] and Lei et al. [52] have characterized human monoclonal antibodies (2H08, 3H03 for N1; 3C08, 3A10, 1F04 for N2) that target these highly conserved dark side epitopes. These regions exhibit spatial conservation across subtypes despite sequence divergence, with similar antigenic architecture observed in both N1 and N2 neuraminidases. The broadly reactive antibodies inhibit NA enzyme activity primarily through steric hindrance, effectively blocking the egress of newly formed viruses from host cells and providing heterosubtypic protection against both seasonal and zoonotic HxN1 strains in animal models [48]. Targeting these conserved regions through precise antigen design offers a unique opportunity to elicit heterosubtypic immunity, providing a level of cross-protection against evolving clades that is largely absent in traditional HA-focused approaches.

Beyond structural conservation, the evolutionary profile of NA provides a compelling strategic advantage, as its rate of antigenic drift is slower compared to that of HA [57]. Crucially, since antibodies against HA and NA independently contribute to protection, targeting both may provide superior defense by limiting immune escape. High-resolution antigenic mapping has confirmed that antigenic drift occurs in a discordant manner for HA and NA [58]. This independent evolution means that genetic changes in HA do not always correspond to changes in NA, and vice versa. Therefore, a vaccine relying solely on HA is more vulnerable to viral escape if that single antigen undergoes a major shift, whereas a bivalent approach ensures a “second line” of immune pressure that is more stable over time.

The theoretical benefits of this second line of defense translate into significant clinical relevance. A recent global expert consensus (Delphi study) concluded that anti-NA responses acquired via vaccination are associated with protective immunity independently of HA [59]. This is further supported by evidence showing that higher levels of pre-existing NA antibodies are associated with reduced symptom duration and decreased viral shedding in naturally infected individuals [60]. Moreover, NA-specific immunity has been shown to reduce overall infectivity, meaning immunized individuals are significantly less likely to transmit the virus within a population [61]. Consequently, targeting NA provides a layer of public health protection, specifically the interruption of transmission chains, that is largely absent in HA-only formulations.

Despite these clear benefits, several “knowledge gaps” continue to stall industry progress, most notably the lack of standardized potency assays and the need for a defined regulatory correlate of protection [62]. Current seasonal mRNA influenza vaccines in late-stage clinical trials continue to focus exclusively on the HA antigen [63], reflecting current licensure requirements that do not mandate NA standardization. Nevertheless, clinical proof-of-concept for a dual-antigen approach was recently demonstrated in a Phase 1/2 randomized trial (NCT05333289) evaluating mRNA vaccines co-encoding seasonal influenza HA and NA antigens (mRNA-1020 and mRNA-1030) in 565 healthy adults aged 18–75 years [64]. The two candidates tested—mRNA-1020 and mRNA-1030—differed in their HA:NA mass ratios (1:1 or 3:1, respectively), an approach designed to explore how closely mimicking the natural stoichiometry of influenza surface glycoproteins influences immunogenicity. Both candidates elicited robust HA-specific immune responses while simultaneously inducing NA-specific immunity, with no vaccine-related serious adverse events and no additional reactogenicity compared to an HA-only mRNA vaccine over 181 days of follow-up. Critically, this was achieved without compromising immunogenicity against either antigen, providing the first human evidence that the mRNA platform can simultaneously encode and co-present both major influenza surface glycoproteins—a task that has proven technically and formulation-wise challenging for conventional egg- and cell-based platforms. Innovative platforms are now also leveraging advanced RNA technologies to address these gaps. For example, bicistronic saRNA allows for the simultaneous encoding of both HA and NA in a single vector [65]. Parallel efforts have led to the development of bivalent mRNA-LNP formulations specifically tailored to the H5N1 clade 2.3.4.4b. A vaccine targeting the human-isolated A/Texas/37/2024 strain has demonstrated 100% protection against lethal challenge in murine models [66].

As the evidence continues to accumulate, there is an increasing consensus around recognizing NAI titers as an independent, secondary correlate of protection in pandemic preparedness [59,60,67]. This should be considered as a new licensure requirement for the next generation influenza vaccines [68]. Leveraging the flexibility of RNA platforms to target conserved epitopes, such as those identified via the CAV-F6 antibody, represents a strategic opportunity to develop more durable, broadly protective, and transmission-blocking vaccines against emerging influenza threats.

### 2.4. Biosecurity and Dual-Use Governance in mRNA-Based Pandemic Preparedness

The same rapid design and manufacturing capabilities that make mRNA platforms strategically valuable for pandemic preparedness also raise important biosecurity and dual-use considerations. The ability to synthesize and deploy antigen-encoding sequences within days of pathogen identification—including sequences derived from highly pathogenic strains such as H5N1 clade 2.3.4.4b—demands robust governance frameworks to prevent misuse and ensure responsible implementation.

Establishing sequence-level oversight mechanisms must be established for select agent-derived genetic material, in alignment with the Biological Weapons Convention (BWC) and WHO guidance on laboratory biosafety and biosecurity [69,70]. The WHO’s Global Guidance Framework for Responsible Life Sciences and Dual-Use Research Governance provides concrete tools and institutional mechanisms—including biorisk assessment protocols, institutional biosafety committee functions, and dual-use research oversight procedures—designed for Member States and research institutions to translate these principles into operational practice [71].

Integrating institutional biorisk assessment into antigen design workflows from the earliest stages, particularly when working with sequences from HPAI strains with documented pandemic potential [72]. Embedding access control and chain-of-custody procedures for synthetic nucleic acid intermediates throughout the manufacturing process, from sequence selection to drug substance production, requires coordination with the U.S. government’s 2023 Screening Framework Guidance, which establishes updated standards for sequence screening and expands the definition of “sequences of concern” to include any sequence contributing to pathogenicity or toxicity, establishing shared responsibility between providers and users [73]. Coordination with suppliers of synthetic DNA and RNA precursors is essential, as many have implemented voluntary screening mechanisms but lack uniform, binding standards. Alignment with international standards for synthetic biology oversight—including frameworks developed by the World Economic Forum and Nuclear Threat Initiative (NTI)—provides an essential reference for responsible governance at both institutional and national levels [74].

These considerations are particularly salient for LMIC-based manufacturers newly entering mRNA production through technology transfer programmes, where biosecurity infrastructure and regulatory oversight capacity may still be maturing. Rather than constituting barriers to technology access, these governance requirements should be viewed as integral components of capacity building—ensuring that the expanded manufacturing capabilities enabled by mRNA technology transfer contribute to global health security without inadvertently increasing dual-use risks. Embedding biosecurity principles into the design of technology transfer programmes from the outset, rather than as a downstream regulatory requirement, represents both a scientific and ethical imperative for next-generation pandemic preparedness.

## 3. Local Experience

### 3.1. Role of Academia–Industry Collaboration in Pandemic Preparedness

Pandemic preparedness relies not only on scientific excellence or manufacturing capacity in isolation, but critically on the strength and maturity of collaborations between academia and industry. The COVID-19 pandemic demonstrated that when these sectors align around shared objectives, vaccine development timelines can be dramatically shortened. Furthermore, technical risks are substantially reduced, as this synergy allows teams to quickly filter out unviable candidates and overcome developmental roadblocks during early proof-of-concept assays. A paradigmatic example is the University of Oxford–AstraZeneca collaboration, where academic development of a viral vector platform was rapidly translated into large-scale manufacturing and global deployment through industrial capabilities [75,76]. As the threat of highly pathogenic avian influenza H5N1 persists, reinforcing and diversifying such collaborative models remains a cornerstone of effective preparedness.

The collaboration models described here were derived from a qualitative synthesis of the literature on vaccine R&D partnerships, particularly studies describing knowledge-sharing and material-transfer dynamics [18,75,76,77,78,79,80], combined with the authors’ experience in academia–industry initiatives in Argentina in the context of pandemic preparedness. To improve clarity and reproducibility, these models are characterized according to key operational dimensions including governance, knowledge transfer, risk sharing, intellectual property, funding, regulatory responsibility, and manufacturing roles (Table 2).

Academia plays a critical role as an early generator of knowledge, often initiating vaccine-related development efforts well before a commercial market exists. These contributions span pathogen-specific vaccine candidates, strategies targeting entire pathogen families, and flexible platform technologies—such as viral vectors and nucleic acid-based approaches—that can be rapidly adapted to emerging threats. Academic institutions contribute deep scientific expertise, conceptual innovation, and flexibility, enabling exploration of high-risk ideas that are often beyond the scope of immediate private-sector investment [18,80].

**Figure 2:**
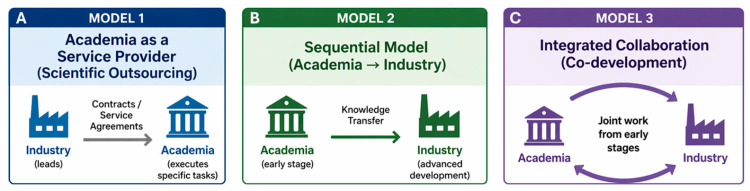
Three models of academia–industry interaction in vaccine development. (**A**) Service provider model. (**B**) Technology transfer from academia to industry model. (**C**) Co-development model with rounds of mutual interaction between partners.

A first, well established interaction model involves academia acting as a specialized scientific service provider for industry (Figure 2A). In this framework, companies outsource defined activities to academic or public research laboratories, such as antigen design, gene optimization, analytical method development, in vitro characterization, animal studies, or early immunogenicity assessments. These collaborations are typically structured through service contracts or targeted research agreements and benefit from the existence of a broad and robust scientific ecosystem. Industry gains access to highly specialized expertise and infrastructure, while academic laboratories receive financial support that sustains research activities and human resource development. Although knowledge generation may remain project-specific, the capabilities developed can ultimately strengthen the broader national innovation system [78].

A second interaction model involves research programs initiated within academia, where early discovery work, including target selection, preliminary production strategies, and initial immunogenicity studies are conducted (Figure 2B). At a subsequent phase, the private sector becomes engaged to advance the candidate from early proof-of-concept into industrial development. This staged model reflects longstanding patterns in vaccine innovation, in which public-sector research de-risks early science and industry assume responsibility for advanced development, large-scale trials, manufacturing, and commercialization. The industrial phase typically encompasses Chemistry, Manufacturing and Controls (CMC) development, analytical validation, stability studies, and coordinated preclinical and clinical execution under regulatory oversight [18,76].

More integrated collaborations represent a third model (Figure 2C), in which academia and industry jointly define projects and share risks, responsibilities, and benefits from early stages onward. These partnerships require mutual alignment: academia must recognize industrial needs related to timelines, reproducibility, and regulatory standards, while industry must acknowledge academia’s priorities including knowledge generation, publication, and training of human resources. In these arrangements, academia contributes specialized expertise—such as deep knowledge of disease biology, antigen design, and advanced analytical techniques—while industry provides experience in industrialization, quality systems, regulatory affairs, and sustained financial support [78,79].

These public–private interactions are particularly critical in low- and middle-income countries where private R&D investment is limited, companies are smaller, and yet scientific systems may be relatively strong. In such contexts, collaboration becomes essential to bridge the persistent gap between basic research and practical application. Argentina illustrates this dynamic through multiple experiences linking public research institutions, universities, and biotechnology companies.

In the specific context of pandemic preparedness, the collaboration between Sinergium Biotech and the Laboratory of Nucleic Acid Immunotechnology (LITAN) at the University of Buenos Aires illustrates an effective academia–industry partnership focused on H5N1 vaccine candidates. Supported by the World Health Organization and funded by the Coalition for Epidemic Preparedness Innovations (CEPI), with coordination support from the Medicines Patent Pool (MPP), this initiative integrates LITAN’s expertise in antigen design, mRNA sequence development, and immunogenicity studies with Sinergium’s capabilities in process design, mRNA production, scale-up, and analytical characterization. This collaboration demonstrates how expertise initially developed around locally relevant diseases can be rapidly adapted to global threats when embedded in well-structured collaborative frameworks [79].

Ultimately, successful academia–industry collaboration depends not only on funding and infrastructure, but on trust, flexibility, and sustained commitment. In the inherently high-risk domain of pandemic preparedness—where products are often developed in advance of defined markets—such collaborations are not optional but strategic necessities for both global and local health security.

### 3.2. Development of Industrial Capabilities Within the mRNA Technology Transfer Programme

Since 2009, Argentina’s private sector has invested strategically in the development of domestic influenza vaccine manufacturing capacity. Sinergium Biotech project emerged in the aftermath of the 2009 H1N1 influenza pandemic, which exposed critical vulnerabilities in the country’s access to vaccines and highlighted the need for local production capabilities. Initially focused on fill-and-finish operations, the project was conceived with a phased approach to progressively incorporate drug substance manufacturing. Sinergium Biotech currently supplies seasonal influenza vaccines to Argentina’s Ministry of Health and, since 2021, has also provided trivalent inactivated influenza vaccines (TIV) through the Pan-American Health Organization (PAHO) Revolving Fund to countries in the Americas. Building on this foundation, the company was selected as a partner for the WHO and MPP co-led mRNA Technology Transfer Programme, a global effort to decentralize vaccine production in the Global South [81].

The company’s strategy is structured around three core pillars: receipt of technology transfer from WHO/MPP, construction of dedicated RNA development and manufacturing facilities, and expansion of the platform toward new vaccine and therapeutic targets.

- Pillar 1: The technology transfer process began in mid-2024 and was executed during October–November 2025.

Familiarization runs with the technology were conducted, including the production of encapsulated mRNA at scales from 0.02 to 1.0 mL of in vitro transcription (IVT) and analytical characterization with a reduced set of assays. Subsequently, the scale was increased to 20 mL and 100 mL of IVT, incorporating technologies such as tangential flow filtration (TFF) and higher-capacity chromatography equipment. The technical team received in-person training at Afrigen (Cape Town, South Africa), and the knowledge acquired was applied at Sinergium Biotech for the production of a technical batch at a 100 mL scale. During this stage, the transferred analytical methods were pre-implemented.

Formal analytical transfer with 18 characterization assays for drug substance and drug product was executed and results were evaluated by MPP/WHO and Afrigen. Finally, the demonstration batch of mRNA/LNP was performed during November 2025 consolidating local mRNA/LNP production capacity in Argentina.

- Pillar 2: Construction of the mRNA development and production plant.

The infrastructure project, initiated in late 2023, was structured into two sequential stages. Stage 1 comprises the construction of the primary building shell and manufacturing areas designed to support research and development as well as scale-up activities. In Stage 2, these areas will be upgraded to comply with Good Manufacturing Practice (GMP) requirements. Completion of Stage 1 is anticipated in the second quarter of 2026. Subsequent activities will include equipment installation, area qualification, and initial validation activities planned for the second half of 2026.

The facility covers approximately 840 m^2^ and is designed to support a 10 L IVT batch process, with an estimated annual output of 15 million doses of 50 µg per dose under a single-shift operation.

- Pillar 3: Expansion of the platform to new vaccine and therapeutic targets.

Since 2024, efforts have focused on leveraging the mRNA platform to address new vaccine and therapeutic targets. Significant progress has been made in designing over a dozen influenza targets, as well as in producing and evaluating encapsulated mRNA in vitro and in vivo, confirming both protein expression and immunogenic potential. Parallel exploratory work has begun on other respiratory, emerging, antimicrobial-resistant and neglected pathogens, along with mRNA-based therapeutics, ensuring the platform’s long-term expansion and sustainability.

## 4. Regional and Global Alignment: How Latin American Experience Integrates into Global Preparedness

Argentina’s experience, together with Sinergium Biotech’s participation in the WHO/MPP mRNA Technology Transfer Programme, illustrates how regional manufacturing initiatives can be effectively aligned with global influenza preparedness objectives while addressing the structural gaps identified by the Global Action Plan for Influenza Vaccines. As highlighted by the GAP experience, the expansion of pandemic-oriented manufacturing capacity without inter-pandemic sustainability, technological flexibility, and integration into broader innovation ecosystems limits long-term preparedness [81]. These lessons are particularly relevant in the context of ongoing H5N1 threats, where the timely availability of vaccines remains uncertain for many low- and middle-income regions.

In this setting, the mRNA platform provides a strategic opportunity to link pandemic influenza preparedness with portfolio diversification. Argentina and Sinergium Biotech have pursued an approach that combines influenza-specific readiness, including the potential rapid development of H5N1 vaccines, with the expansion of the platform toward seasonal influenza, other respiratory pathogens, and RNA-based therapeutics. The inclusion of RNA-based therapeutics is especially relevant, as this field continues to expand within the biopharmaceutical market and offers opportunities to sustain manufacturing activity, workforce expertise, and technological development beyond vaccine-only applications. Such diversification directly addresses one of the central limitations observed under GAP, namely the erosion of capacity during inter-pandemic periods. In addition to product diversification, the provision of contract development and manufacturing services (CDMO/CMO) represents a complementary strategy to sustain mRNA manufacturing facilities during inter-pandemic periods. By operating as contract manufacturers, regional facilities can produce mRNA batches for preclinical and clinical development programs led by international biotechnology companies that lack in-house GMP manufacturing capabilities. This model not only generates alternative revenue streams, but also contributes to maintaining operational readiness, workforce expertise, and quality systems in continuous use, thereby reducing the risk of infrastructure underutilization between pandemic events. Moreover, Sinergium Biotech has been investing in high formulation and fill/finish capacity through its partnerships with multinational companies to supply the region with vaccines from Argentina. This capacity in vials and pre-filled syringes of more than 450 million doses/year is also a fundamental pillar for pandemic response in the region and eventually, beyond.

At the same time, the translation of distributed manufacturing capacity into public health impact depends on alignment with global and regional procurement mechanisms. Commitments from multilateral organizations, including PAHO, to support vaccine purchasing during a pandemic are essential to ensure predictable demand and enable regional manufacturers to rapidly pivot toward pandemic influenza vaccine production when needed. In this context, Argentina’s experience with PAHO procurement mechanisms for seasonal influenza vaccines provides a relevant foundation for future pandemic scenarios.

Beyond procurement alignment, the long-term viability of regional mRNA manufacturing depends on a set of economic and structural conditions that must be deliberately designed rather than assumed. First, the product portfolio must extend beyond pandemic influenza to encompass seasonal vaccines, other infectious diseases, and RNA-based therapeutics. A critical lesson underscored by current WHO-supported initiatives exploring dengue, HPV, malaria, and other vaccine targets to sustain inter-pandemic operations [82,83]. Indeed no single pandemic-use product can sustain a manufacturing facility through inter-pandemic periods, and the WHO’s recent emphasis on portfolio diversification reflects the recognition that manufacturers must develop “commercially viable products that they can sell and that there are people out there who want these products” [84]. A complementary dimension supporting the sustainability of mRNA manufacturing is its potential application in veterinary vaccinology [85,86,87]. The development and production of mRNA-based vaccines for animal health including poultry [85], livestock [88], and zoonotic disease reservoirs [89] represent an additional and underexplored source of stable demand.

Second, the cost structure of mRNA production, while more adaptable than egg-based platforms, still requires stable capital investment in infrastructure, cold chain logistics, and specialized analytical capacity [90,91]. In addition, current mRNA manufacturing processes are associated with relatively high cost of goods (COGs), driven by the need for specialized raw materials such as modified nucleosides, capping reagents, and lipid nanoparticle components, as well as complex quality control requirements. Without predictable demand signals from procurement bodies such as PAHO or COVAX successor mechanisms, this investment remains financially precarious for manufacturers in LMICs.

Third, supply chain resilience requires regionalization of key inputs—including lipid components for LNP formulation, capping enzymes, and nucleoside-modified precursors—many of which are currently sourced from a small number of suppliers concentrated in high-income countries.

A disruption in any of these inputs during a pandemic scenario could neutralize the speed advantage that mRNA platforms are expected to provide. Addressing these vulnerabilities requires not only industrial investment, but coordinated policy action, including advanced purchase commitments, regional supply chain mapping, and integration of mRNA manufacturing into national strategic stockpiling frameworks.

Taken together, the experience of Argentina and Sinergium Biotech demonstrates how regionally embedded and technologically flexible manufacturing platforms, supported by diversified product portfolios and aligned with global purchasing strategies, can strengthen preparedness for influenza pandemics, including H5N1. More broadly, it underscores that equitable global pandemic preparedness requires not only expanded manufacturing capacity, but also sustained and integrated global–regional strategies that connect technology transfer, market design, and long-term public health priorities.

## 5. Conclusions

The ongoing global spread of highly pathogenic avian influenza H5N1 emphasizes the urgent need to advance pandemic preparedness beyond traditional, monovalent vaccine paradigms. While past initiatives have expanded manufacturing capacity and improved global coordination, persistent gaps remain in the sustainability, flexibility, and equitable distribution of vaccine production, particularly in low- and middle-income countries (LMICs). This perspective highlights the convergence of three critical dimensions required to address these challenges. First, mRNA platforms provide a transformative technological foundation, enabling rapid vaccine design, scalable manufacturing, and adaptability across multiple pathogens. However, this power must be paired with a strict biorisk assessment and dual-use governance procedures to ensure secure and responsible development. Second, advances in antigen design, including the incorporation of neuraminidase as a complementary target, offer the potential to enhance the breadth and durability of immune protection. Third, the development of regionally embedded manufacturing systems—supported by initiatives such as the mRNA Technology Transfer Programme co-led by the World Health Organization (WHO) and the Medicines Patent Pool (MPP)—demonstrates how global strategies can be translated into sustainable local capabilities.

The experience of Argentina and Sinergium Biotech illustrates how these elements can be integrated into a coherent model for pandemic preparedness, combining technological innovation, academia–industry collaboration, and strategic alignment with global procurement mechanisms. Importantly, this model addresses key limitations identified in previous initiatives, particularly the need for diversification and economic viability during inter-pandemic periods.

Looking forward, effective preparedness will require not only continued investment in advanced vaccine technologies, but also coordinated efforts to align technology transfer, manufacturing capacity, and market-shaping mechanisms at both global and regional levels. Building resilient, adaptable, and sustainable vaccine ecosystems is essential to ensure that future pandemic responses are both rapid and equitable.

## Figures and Tables

**Table 1:** From GAP Failures to mRNA Solutions: A Structural Contrast.

Challenge Under GAP (Egg-Based Platforms)	How mRNA Platforms Address It
Viability depended on seasonal influenza vaccine demand, which was insufficient to sustain facilities in LMICs inter-pandemically	mRNA facilities can pivot to other antigens (e.g., rabies, oncology, other respiratory viruses), enabling a diversified product portfolio that supports year-round operations
Long lead times due to specialized egg supply chains and biological amplification constraints	mRNA synthesis is cell-free and antigen-agnostic; once a sequence is available, manufacturing can begin within weeks
Platform locked to a single pathogen, limiting commercial and public health flexibility	The same platform and infrastructure can produce vaccines against multiple pathogens, reducing dependency on pandemic-driven demand
Technology transfer required replication of complex biological processes, limiting scalability	mRNA manufacturing is modular and amenable to standardized, transferable protocols—a key advantage for regional technology transfer programs
Infrastructure erosion during inter-pandemic periods due to lack of utilization	A diversified pipeline sustains workforce expertise, supply chain readiness, and regulatory capacity continuously

**Table 2:** Operational framework for academia–industry collaboration models in vaccine development.

Collaboration Model	Governance	Knowledge Transfer	Risk Sharing	Intellectual Property	Funding	Regulatory Responsibility	Manufacturing Role
Service-based (Academia as provider)	Industry-led	Unidirectional (task-specific)	Low (industry assumes main risk)	Typically retained by industry	Industry-funded (contract-based)	Industry	Industry
Sequential (Academia → Industry)	Staged/sequential	Transfer from academia to industry	Shared (early risk in academia, late-stage risk in industry)	Transferred or licensed	Mixed (public early, private later)	Industry (later stages)	Industry
Integrated collaboration	Joint governance	Bidirectional (co-development)	Shared across partners	Shared or negotiated	Mixed (public + private)	Shared/coordinated	Industry (with academic input)

## Data Availability

The data presented in this study are available on the Protein Data Bank at RCSB PDB: Homepage, PDB reference number, 8E6J and 8EZ3.

## Abbreviations

The following abbreviations are used in this manuscript:

GAP	Global Action Plan
GMP	Good Manufacturing Practice
HA	Hemagglutinin
HPAI	Highly Pathogenic Avian Influenza A
IVT	In Vitro Transcription
LMICs	Low- and Middle-Income Countries
MPP	Medicines Patent Pool
mRNA	Messenger RNA
NA	Neuraminidase
ORF	Open Reading Frame
PAHO	Pan-American Health Organization
saRNA	Self-amplifying RNA
TFF	Tangential Flow Filtration
WHO	World Health Organization
